# Association of Hepatorenal Syndrome-Acute Kidney Injury with Mortality in Patients with Cirrhosis Requiring Renal Replacement Therapy: Results from the HRS-HARMONY Consortium

**DOI:** 10.34067/KID.0000000589

**Published:** 2024-09-30

**Authors:** Augusto Cama-Olivares, Tianqi Ouyang, Tomonori Takeuchi, Shelsea A. St. Hillien, Jevon E. Robinson, Raymond T. Chung, Giuseppe Cullaro, Constantine J. Karvellas, Josh Levitsky, Eric S. Orman, Kavish R. Patidar, Kevin R. Regner, Danielle L. Saly, Deirdre Sawinski, Pratima Sharma, J. Pedro Teixeira, Nneka N. Ufere, Juan Carlos Q. Velez, Hani M. Wadei, Nabeel Wahid, Andrew S. Allegretti, Javier A. Neyra, Justin M. Belcher

**Affiliations:** 1 Division of Nephrology, Department of Medicine, University of Alabama at Birmingham, Birmingham, Alabama; 2 Division of Nephrology, Department of Medicine, Massachusetts General Hospital, Boston, Massachusetts; 3 Department of Health Policy and Informatics, Graduate School of Medical and Dental Sciences, Tokyo Medical and Dental University, Tokyo, Japan; 4 Division of Gastroenterology, Department of Medicine, Liver Center, Massachusetts General Hospital, Boston, Massachusetts; 5 Division of Gastroenterology and Hepatology, Department of Medicine, University of California, San Francisco, California; 6 Division of Gastroenterology (Liver Unit), Department of Critical Care Medicine, University of Alberta, Edmonton, Alberta, Canada; 7 Comprehensive Transplant Center, Northwestern University Feinberg School of Medicine, Chicago, Illinois; 8 Division of Gastroenterology and Hepatology, Department of Medicine, Northwestern University Feinberg School of Medicine, Chicago, Illinois; 9 Division of Gastroenterology and Hepatology, Indiana University School of Medicine, Indianapolis, Indiana; 10 Section of Gastroenterology and Hepatology, Department of Medicine, Baylor College of Medicine and Michael E. DeBakey Veterans Affairs Medical Center, Houston, Texas; 11 Division of Nephrology at the Medical College of Wisconsin, Medical College of Wisconsin, Milwaukee, Wisconsin; 12 Division of Nephrology and Hypertension, Weill Cornell College of Medicine, New York, New York; 13 Department of Gastroenterology and Transplant Hepatology at University of Michigan Health, University of Michigan Health, Ann Arbor, Michigan; 14 Divisions of Nephrology and Pulmonary, Critical Care, and Sleep Medicine, Department of Internal Medicine, University of New Mexico, Albuquerque, New Mexico; 15 Department of Nephrology at the Ochsner Medical Center, Ochsner Medical Center, New Orleans, Louisiana; 16 Department of Transplantation, Mayo Clinic, Jacksonville, Florida; 17 Section of Nephrology, Department of Internal Medicine, Yale University and VA Connecticut Healthcare, New Haven, Connecticut

**Keywords:** acute kidney failure, AKI, dialysis, hemodialysis, hospitalization, liver failure, mortality, outcomes, renal dialysis

## Abstract

**Key Points:**

- In patients with cirrhosis and AKI requiring renal replacement therapy (RRT), hepatorenal syndrome-AKI was not associated with an increased 90-day mortality when compared with other AKI etiologies.
- Etiology of AKI may not be a critical factor regarding decisions to trial RRT in acutely ill patients with cirrhosis and AKI.
- Although elevated, mortality rates in this study are comparable with those reported in general hospitalized patients with AKI requiring RRT.

**Background:**

While AKI requiring renal replacement therapy (AKI-RRT) is associated with increased mortality in heterogeneous inpatient populations, the epidemiology of AKI-RRT in hospitalized patients with cirrhosis is not fully known. Herein, we evaluated the association of etiology of AKI with mortality in hospitalized patients with cirrhosis and AKI-RRT in a multicentric contemporary cohort.

**Methods:**

This is a multicenter retrospective cohort study using data from the HRS-HARMONY consortium, which included 11 US hospital network systems. Consecutive adult patients admitted in 2019 with cirrhosis and AKI-RRT were included. The primary outcome was 90-day mortality, and the main independent variable was AKI etiology, classified as hepatorenal syndrome (HRS-AKI) versus other (non–HRS-AKI). AKI etiology was determined by at least two independent adjudicators. We performed Fine and Gray subdistribution hazard analyses adjusting for relevant clinical variables.

**Results:**

Of 2063 hospitalized patients with cirrhosis and AKI, 374 (18.1%) had AKI-RRT. Among them, 65 (17.4%) had HRS-AKI and 309 (82.6%) had non–HRS-AKI, which included acute tubular necrosis in most cases (62.6%). Continuous renal replacement therapy was used as the initial modality in 264 (71%) of patients, while intermittent hemodialysis was used in 108 (29%). The HRS-AKI (versus non–HRS-AKI) group received more vasoconstrictors for HRS management (81.5% versus 67.9%), whereas the non–HRS-AKI group received more mechanical ventilation (64.3% versus 50.8%) and more continuous renal replacement therapy (versus intermittent hemodialysis) as the initial RRT modality (73.9% versus 56.9%). In the adjusted model, HRS-AKI (versus non–HRS-AKI) was not independently associated with increased 90-day mortality (subdistribution hazard ratio, 1.36; 95% confidence interval, 0.95 to 1.94).

**Conclusions:**

In this multicenter contemporary cohort of hospitalized adult patients with cirrhosis and AKI-RRT, HRS-AKI was not independently associated with an increased risk of 90-day mortality when compared with other AKI etiologies. The etiology of AKI appears less relevant than previously considered when evaluating the prognosis of hospitalized adult patients with cirrhosis and AKI requiring RRT.

## Visual Abstract

**Figure:**
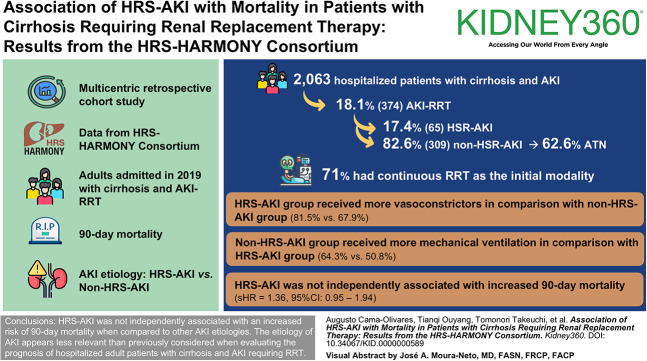

## Introduction

AKI is a common and challenging complication of cirrhosis and acute-on-chronic liver failure (ACLF).^1,2^ It is present in up to 50% of hospitalized patients with cirrhosis,^3–7^ increasing the risk of complications and death.^8–12^ Patients with cirrhosis are at high risk of AKI because of hemodynamic changes, impaired effective circulating volume, and excess vasoconstriction and inflammation.^2,13^ Although hepatorenal syndrome (HRS-AKI) is one of the most severe etiologies of AKI in patients with decompensated cirrhosis,^4,14^ there are many other potential causes, such as sepsis, intra-abdominal hypertension, and nephrotoxins, capable of inducing tubular damage and acute dysfunction, aggregated here as non–HRS-AKI.^15–17^ HRS-AKI and non–HRS-AKI are often accompanied by certain pathobiologic differences that may affect prognosis. For instance, patients with HRS-AKI are at higher risk of cardiac dysfunction and adrenal insufficiency because of systemic vasoconstriction.^15^

The use of requiring renal replacement therapy (RRT) in patients with cirrhosis and AKI has increased.^18,19^ Approximately 20%–40% of critically ill patients with cirrhosis and AKI require RRT.^18,20^ However, there is a lack of information about best practices for the management of AKI with RRT in this setting and uncertainty as to relevant or potentially modifiable factors that influence prognosis. RRT is traditionally recommended as a bridge to liver transplantation, but its utilization in nontransplant candidates remains controversial and is often only recommended in patients with an acute but reversible decompensation.^2,13,21^ RRT modality is generally determined by the hemodynamic stability and severity of illness of the patient.^22–24^ In patients with cirrhosis and AKI requiring RRT (AKI-RRT), the use of continuous RRT (CRRT), compared with intermittent hemodialysis (IHD), has been associated with a higher risk of mortality, but these results may be biased by the overall higher acuity of illness status of patients receiving CRRT.^11,25,26^

Previous studies have shown mortality differences regarding the etiology of AKI in patients with cirrhosis across a range of AKI severity,^14,27,28^ but these differences were not found in single-center studies that evaluated only patients with cirrhosis and AKI-RRT.^29,30^ Nevertheless, available literature about the impact of the etiology of AKI on mortality, particularly when differentiating HRS versus non–HRS, is limited by small sample size, older studies, or single-center examinations that do not represent contemporary practice. In this study, we examined the clinical characteristics of patients with cirrhosis and AKI-RRT, stratified by AKI etiology, and the association of AKI etiology (HRS-AKI versus non–HRS-AKI) with 90-day mortality in a large multicenter contemporary cohort.

## Methods

### Study Design and Population

This multicenter retrospective cohort study uses data from the HRS-HARMONY consortium, which included hospitalized patients with AKI and cirrhosis in 11 US hospital network systems (11 liver transplant centers and 15 hospitals) from January 2019 to December 2019. Consecutive hospitalized adult (≥18 years) patients with cirrhosis and AKI-RRT were included.

Details of the HRS-HARMONY cohort have been previously published.^14^ Briefly, the HRS-HARMONY cohort included patients with at least one International Classification of Diseases Tenth Revision code of cirrhosis and an inpatient serum creatinine >1.5 mg/dl. Subsequently, the accuracy of the diagnoses was validated by manual chart review for final eligibility. The HRS-HARMONY cohort excluded patients with liver disease without cirrhosis, those who did not meet criteria for AKI, had prior liver or kidney transplant, received RRT at the time of admission, had AKI only after liver transplantation, or with incomplete clinical data. The data were collected from electronic health records, United Network for Organ Sharing records, and online obituaries and/or the Social Security Death Index.

### Study Variables and Definitions

The diagnosis and etiology of cirrhosis were determined based on the reported clinical judgment of the treating hepatologist and confirmed by radiologic evidence and presence of complications related to cirrhosis, liver biopsy, and/or endoscopic evidence of portal hypertension. Patients active on the United Network for Organ Sharing waiting list during the index admission were classified as listed for liver transplant.

AKI was defined using the 2015 International Club of Ascites-AKI criteria: an increase in serum creatinine ≥0.3 mg/dl within 48 hours or a percentage increase in serum creatinine ≥50% from baseline.^31^ The baseline serum creatinine was recorded as the most recent outpatient value within 1 year before the index admission. In the HRS-HARMONY cohort, the etiology of AKI was divided into four categories (pre-renal, HRS-AKI, acute tubular necrosis [ATN], and other) and determined on retrospective chart review by at least two independent adjudicators, with a third included if needed. The reviewers classified the patient as HRS-AKI if the patient met the 2015 International Club of Ascites HRS-AKI definition.^31^ The categories of AKI etiology were mutually exclusive, and patients were deemed as unable to be classified if there was a disagreement between the three adjudicators or insufficient clinical information. In this study, the etiology of AKI was classified into two categories: HRS-AKI and all other etiologies (pre-renal, ATN, others, and unable to be classified), referred to as non–HRS-AKI. This study adhered to the recommendations of the Acute Dialysis Quality Initiative 29th conference.^32^ All other clinical data, including the initial RRT modality (CRRT versus IHD), were accessed from electronic health records. Prolonged intermittent RRT was collected as part of the CRRT category.

The RRT volume capacity of study sites was classified according to the number of RRT machines. Study sites with >20 hemodialysis machines and >20 CRRT machines were classified as high-volume RRT centers. The other study sites were classified as non–high-volume RRT centers. This information, specifically RRT capacity, was collected through a survey tool sent to each study site investigator and is reported in Supplemental Table 1.

Variables related to clinical processes, such as intensive care unit (ICU) admission, vasopressors for shock, vasoconstrictors for HRS, and mechanical ventilation, were assessed during the entire hospitalization. Therefore, some patients with HRS-AKI may have developed shock requiring vasopressors after the diagnosis of HRS-AKI was made.

### Outcomes and Statistical Analysis

Patient characteristics and study variables are presented as medians/means and interquartile ranges/SDs for continuous variables and percentages for categorical variables. Characteristics of the cohort were compared using Student *t* test or Wilcoxon rank sum for continuous variables and Chi-square or Fisher exact test for categorical variables, as appropriate. The primary study outcome was all-cause 90-day mortality, and the main exposure (independent variable) was the etiology of AKI (HRS-AKI versus non–HRS-AKI).

Cumulative incidence curves for 90-day mortality were estimated by AKI etiology using the Nelson Aalen estimator, with liver transplantation considered a competing risk. A prespecified Fine and Gray subdistribution hazard model, accounting for competing risks of liver transplant,^33^ was constructed using the previous literature, clinical rationale, and results of univariate analyses.^27–29^ The model was adjusted for age, sex, race, ethnicity, RRT volume capacity of the study site (high-volume RRT versus non–high-volume RRT hospital), exposure to vasopressors for shock, initial RRT modality, model for end-stage liver disease-sodium (MELD-Na) score, and transplant listing status. The interactions between the study site, AKI etiology, and 90-day mortality and between transplant listing status, AKI etiology, and 90-day mortality were evaluated. As part of sensitivity analyses, we first compared HRS-AKI versus ATN, only as reference, and next included the chronic liver failure consortium acute-on-chronic liver failure (CLIF-C ACLF) score in the model and removed the variables age, vasopressor for shock, and MELD-Na score because of collinearity. Statistical analyses and figures were performed with R studio version 4.3.1. Statistical significance was set at *P* = 0.05 (two-tailed).

### Ethics

The study was approved by the Institutional Review Boards of each participating site, with waiver of informed consent given the retrospective nature of the investigation. The study was conducted in accordance with the principles of the Declaration of Helsinki.

## Results

### Study Cohort Characteristics

A total of 374 of 2063 consecutively hospitalized patients with cirrhosis and AKI received RRT and were included in the analysis (Table 1). The median age was 58 (interquartile range [IQR], 48–65) years, 160 (42.8%) were female, and 72.5% were White patients. The most common etiology of cirrhosis was alcohol (41.7%), followed by metabolic dysfunction-associated steatotic liver disease (19.3%) and 24.1% had prevalent CKD. The majority were admitted to the ICU (88.8%), received vasopressors for shock (69.4%), and received mechanical ventilation (61.9%). A total of 29% and 71% received IHD and CRRT as the initial RRT modality, respectively, and the median time from admission to RRT initiation was 4 (IQR, 1–9) days. The median MELD-Na and CLIF-C ACLF scores were 32 (IQR, 25–37) and 56.1 (IQR, 49.1–62.5), respectively. Patients who received CRRT (versus IHD) had a higher median MELD-Na (32 [IQR, 26–34] versus 30 [IQR, 23–35]) and CLIF-C ACLF score (56.9 [IQR, 50.5–62.9] versus 53.8 [IQR, 47.0–59.5]) (Supplemental Table 2).

**Table 1:** Demographics and clinical characteristics of the study cohort by etiology of AKI (HRS-AKI versus non–HRS-AKI)

Parameter	All Patients (*N*=374)	HRS-AKI (*n*=65)	Non–HRS-AKI (*n*=309)	*P* Value
**Demographics**				
Age (yr)	58 (48–65)	58 (48–64)	58 (47–66)	0.62
Female sex, *No. (%)*	160 (42.8)	31 (47.7)	129 (41.8)	0.38
White race, *No. (%)*	271 (72.5)	50 (76.9)	221 (71.5)	0.38
Hispanic ethnicity, *No. (%)*	43 (11.5)	11 (16.9)	32 (10.4)	0.10
**Comorbidities**				
Etiology of cirrhosis, *No. (%)*				0.003
*Alcohol*	156 (41.7)	27 (41.5)	129 (41.8)	
*Hepatitis C*	35 (9.4)	5 (7.7)	30 (9.7)	
*MASLD*	72 (19.3)	23 (35.4)	49 (15.9)	
*Multifactorial*	28 (7.5)	1 (1.5)	27 (8.7)	
*Other*	83 (22.1)	9 (13.9)	74 (23.9)	
Diabetes, *No. (%)*	130 (34.8)	23 (35.4)	107 (34.6)	0.91
Coronary artery disease, *No. (%)*	58 (15.5)	7 (10.8)	51 (16.5)	0.25
CKD, *No. (%)*	90 (24.1)	22 (34.4)	68 (22.0)	0.04
Hypertension, *No. (%)*	170 (45.5)	27 (41.5)	143 (46.3)	0.49
**Complications of cirrhosis, *No.* (%)**				
Ascites	317 (84.8)	63 (96.9)	254 (82.2)	0.003
Encephalopathy	253 (67.8)	44 (67.7)	209 (67.9)	0.98
Gastrointestinal bleeding	147 (39.4)	26 (40.0)	121 (39.3)	0.98
Spontaneous bacterial peritonitis	76 (20.4)	17 (26.2)	59 (19.2)	0.20
Hepatocellular carcinoma	23 (6.2)	5 (7.7)	18 (5.8)	0.37
**Characteristics of admission**				
ICU admission, *No. (%)*	332 (88.8)	55 (84.6)	277 (89.6)	0.24
Admission MAP (mm Hg)	66.0 (57.4–74.0)	70.0 (57.3–75.0)	65.3 (57.7–74.0)	0.30
Vasopressor for shock, *No. (%)*	258 (69.4)	39 (61.9)	219 (70.9)	0.16
Vasoconstrictor for HRS, *No. (%)*	262 (70.2)	53 (81.5)	209 (67.9)	0.03
Albumin given during admission, *No. (%)*	320 (86.3)	62 (96.9)	258 (84.0)	0.007
Total albumin given during admission^a^ (g)	125 (75–200)	125 (97–200)	125 (75–195)	0.23
Mechanical ventilation, *No. (%)*	231 (61.9)	33 (50.8)	198 (64.3)	0.04
Initial RRT modality, *No. (%)*				0.006
*IHD*	108 (29.0)	28 (43.1)	80 (26.1)	
*CRRT*	264 (71.0)	37 (56.9)	227 (73.9)	
Days from admission to RRT initiation^b^	4 (1–9)	5 (2–11)	4 (1–9)	0.15
**Survival scores**				
MELD-Na score	32 (25–37)	33 (27–35)	31 (24–37)	0.27
CLIF-C ACLF score	56.1 (49.1–62.5)	53.6 (47.3–61.5)	56.6 (49.5–62.8)	0.17
**Laboratory values**				
Sodium (mEq/L)	133 (128–137)	132 (127–137)	133 (128–137)	0.18
Admission creatinine (mg/dl)	2.5 (1.5–3.9)	2.8 (1.9–3.9)	2.4 (1.5–3.9)	0.20
Peak creatinine (mg/dl)	4.0 (2.9–5.9)	4.1 (3.2–5.8)	4.0 (2.9–5.8)	0.42
BUN (mg/dl)	44 (28–65)	52 (39–73)	41 (25–63)	0.003
White blood count (K/*μ*l)	10.1 (6.7–16.0)	10.7 (7.7–16.9)	9.9 (6.6–15.5)	0.33
Albumin (g/dl)	2.9 (2.4–3.3)	2.9 (2.5–3.5)	2.9 (2.4–3.3)	0.35
INR	2.0 (1.5–2.7)	1.9 (1.6–2.4)	2.0 (1.5–2.7)	0.67
Total bilirubin (mg/dl)	6.2 (2.2–16.8)	6.6 (3.1–15.6)	6.2 (2.1–17.0)	0.48
FENa^c^, *No. (%)*	0.3 (0.2–0.7)	0.3 (0.1–0.5)	0.4 (0.2–1.0)	0.06

Continuous variables are reported as median (interquartile range). ACLF, acute-on-chronic liver failure; CLIF-C ACLF, chronic liver failure consortium acute-on-chronic liver failure; CRRT, continuous renal replacement therapy; FENa, fractional excretion of sodium; HRS, hepatorenal syndrome; ICU, intensive care unit; IHD, intermittent hemodialysis; INR, international normalized ratio; MAP, mean arterial pressure; MASLD, metabolic dysfunction-associated steatotic liver disease; MELD-Na, model for end-stage liver disease-sodium; RRT, renal replacement therapy.

a Available in 272 patients.

b Available in 333 patients.

c Available in 229 patients.

### Characteristics According to AKI Etiology

**Figure 1:**
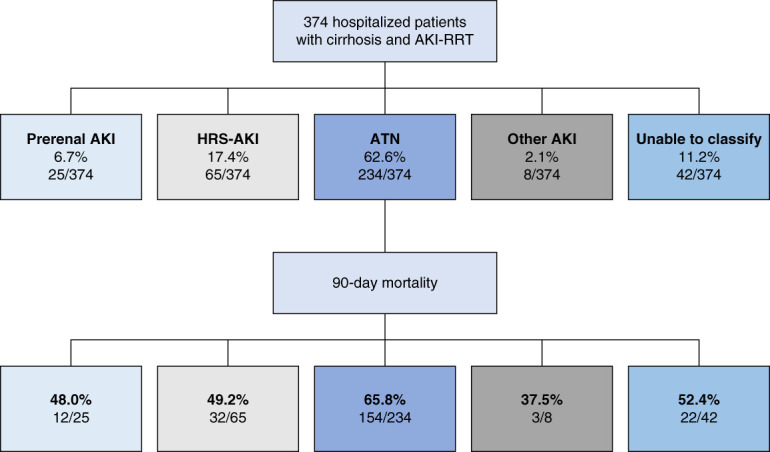
**Distribution and outcomes of the study cohort by etiology of AKI-RRT.** AKI-RRT, AKI requiring renal replacement therapy; ATN, acute tubular necrosis; HRS, hepatorenal syndrome; RRT, renal replacement therapy.

The demographics and clinical characteristics of the study cohort by etiology of AKI are presented in Supplemental Table 3. Overall, 6.7% had prerenal AKI, 17.4% had HRS-AKI, 62.6% had ATN, 2.1% had other AKI etiology, and 11.2% were unable to be classified (Figure 1). Patients in the non–HRS-AKI group received CRRT more frequently as the initial RRT modality than patients with HRS-AKI (73.9% versus 56.9%, *P* = 0.006) (Table 1). The HRS-AKI (versus non–HRS-AKI) group more frequently received HRS-specific vasoconstrictors (81.5% versus 67.9%, *P* = 0.03) and albumin during admission (96.9% versus 84.0%, *P* = 0.007). However, the median cumulative albumin given during admission was comparable between groups (125 [IQR, 97–200] versus 125 [IQR, 75–195] grams, *P* = 0.23, respectively). By contrast, the non–HRS-AKI group more frequently received mechanical ventilation (64.3% versus 50.8%, *P* = 0.04).

### Characteristics According to 90-Day Mortality

The overall 90-day mortality rate of the study cohort was 59.6%. Patient characteristics by vital status at 90 days are presented in Table 2. Patients who died more frequently required ICU admission (93.3% versus 82.1%, *P* = 0.001), vasopressors for shock (77.6% versus 57.0%, *P* < 0.001), and mechanical ventilation (74.8% versus 43.0%, *P* < 0.001). The 90-day mortality rate was higher in patients who received vasopressors for shock in both HRS-AKI (59.0% versus 37.5%, *P* = 0.10) and non–HRS-AKI (68.5% versus 45.6%, *P* < 0.001) groups. The 90-day mortality rate was higher in those who initiated RRT with CRRT as compared with IHD (68.9% versus 38.0%, *P* < 0.001). Patients who died had higher CLIF-C ACLF scores than survivors (median 57.3 versus 53.8, *P* < 0.001), but no difference was found in MELD-Na score (*P* = 0.19).

**Table 2:** Patient characteristics of the study cohort by vital status at 90 days

Parameter	Alive (*n*=151)	Dead (*n*=223)	*P* Value
**Demographics**			
Age (yr)	57 (48–65)	59 (48–66)	0.55
Female sex, *No.* (%)	66 (43.7)	94 (42.2)	0.85
White race, *No. (%)*	105 (69.5)	166 (74.4)	0.36
Hispanic ethnicity, *No. (%)*	22 (14.6)	21 (9.4)	0.17
**Comorbidities**			
Etiology of cirrhosis, *No. (%)*			0.22
*Alcohol*	58 (38.4)	98 (43.9)	
*Hepatitis C*	12 (7.9)	23 (10.3)	
*MASLD*	37 (24.5)	35 (15.7)	
*Multifactorial*	9 (6.0)	19 (8.5)	
*Other*	35 (23.2)	48 (21.6)	
Diabetes, *No. (%)*	64 (42.4)	66 (29.6)	0.02
Coronary artery disease, *No. (%)*	25 (16.6)	33 (14.8)	0.75
CKD, *No. (%)*	52 (34.7)	38 (17.0)	<0.001
Hypertension, *No. (%)*	72 (47.7)	98 (43.9)	0.54
**Complications of cirrhosis, No. (%)**			
Ascites	122 (80.8)	195 (87.4)	0.11
Encephalopathy	92 (61.3)	161 (72.2)	0.04
Gastrointestinal bleeding	61 (40.7)	86 (38.6)	0.77
Spontaneous bacterial peritonitis	28 (18.7)	48 (21.5)	0.59
Hepatocellular carcinoma	10 (6.7)	13 (5.8)	0.91
**Characteristics of admission**			
ICU admission, *No. (%)*	124 (82.1)	208 (93.3)	0.001
Admission MAP (mm Hg)	68.3 (58.5–76.3)	64.3 (56.8–72.7)	0.01
Vasopressor for shock, *No. (%)*	85 (57.0)	173 (77.6)	<0.001
Vasoconstrictor for HRS, *No. (%)*	101 (67.3)	161 (72.2)	0.37
Albumin given during admission, *No. (%)*	124 (83.2)	196 (88.3)	0.22
Total albumin given during admission^a^ (g)	125 (75–169)	125 (75–200)	0.43
Mechanical ventilation, *No. (%)*	65 (43.0)	166 (74.8)	<0.001
Initial RRT modality, *No. (%)*			<0.001
*IHD*	67 (45.0)	41 (18.4)	
*CRRT*	82 (55.0)	182 (81.6)	
Days from admission to RRT initiation^b^	4 (2–8)	4 (1–10)	0.97
**Survival scores**			
MELD-Na score	32 (24–36)	32 (25–38)	0.19
CLIF-C ACLF score	53.8 (46.1–59.5)	57.3 (51.0–63.5)	<0.001
**Laboratory values**			
Sodium (mEq/L)	134 (129–138)	133 (128–137)	0.65
Admission creatinine (mg/dl)	2.8 (1.7–4.4)	2.4 (1.3–3.6)	0.006
Peak creatinine (mg/dl)	5.0 (3.4–6.2)	3.8 (2.8–5.2)	0.001
BUN (mg/dl)	48.0 (32.5–71.0)	41.0 (24.0–60.0)	0.004
White blood count (K/*μ*l)	9.4 (6.2–15.2)	10.7 (7.3–16.7)	0.10
Albumin (g/dl)	3.0 (2.6–3.4)	2.8 (2.4–3.2)	0.003
INR	1.8 (1.4–2.3)	2.1 (1.6–2.7)	0.002
Total bilirubin (mg/dl)	4.4 (1.2–13.8)	6.8 (2.8–17.4)	0.004
FENa^c^, *No. (%)*	0.5 (0.2–0.9)	0.3 (0.2–0.6)	0.04

Continuous variables are reported as median (interquartile range). ACLF, acute-on-chronic liver failure; CLIF-C ACLF, chronic liver failure consortium acute-on-chronic liver failure; CRRT, continuous renal replacement therapy; FENa, fractional excretion of sodium; HRS, hepatorenal syndrome; ICU, intensive care unit; IHD, intermittent hemodialysis; INR, international normalized ratio; MAP, mean arterial pressure; MASLD, metabolic dysfunction-associated steatotic liver disease; MELD-Na, model for end-stage liver disease-sodium; RRT, renal replacement therapy.

a Available in 272 patients.

b Available in 333 patients.

c Available in 229 patients.

### Characteristics According to Liver Transplant Listing Status

A total of 99 (26.5%) patients were listed for liver transplant, of whom 64 (17.1%) were transplanted. The 90-day mortality rate was lower in listed patients (28.3% versus 70.9% in those not listed, *P* < 0.001). Comparisons of patient characteristics by liver transplant listing status are presented in Supplemental Table 4. Patients with HRS-AKI more frequently were listed for (47.7% versus 22.0%, *P* < 0.001) and received (36.9% versus 12.9%, *P* < 0.001) liver transplant than patients with non–HRS-AKI. Patients listed for liver transplant had fewer comorbidities, such as coronary artery disease and hypertension. They were also more frequently treated with vasoconstrictors for HRS and albumin during the index admission and had higher MELD-Na scores. Among patients listed for liver transplant, the 90-day mortality rates in patients with HRS-AKI and non–HRS-AKI were 22.6% and 30.9%, respectively (*P* = 0.40). In nonlisted patients, the 90-day mortality rates were similar between patients with HRS-AKI and non–HRS-AKI (73.5% versus 70.5%, *P* = 0.72).

### Association of AKI Etiology with 90-Day Mortality

**Figure 2:**
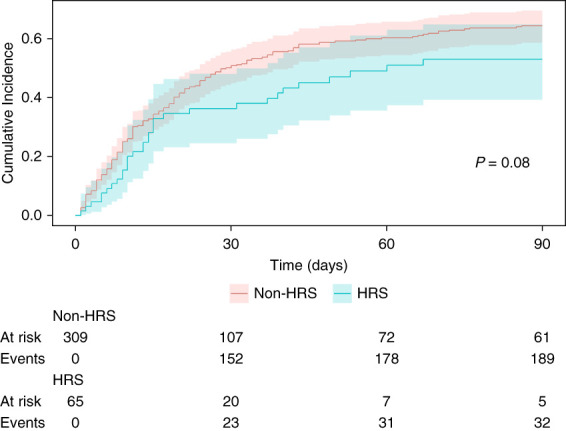
**Nelson Aalen curves showing cumulative incidence of all-cause 90-day mortality by etiology of AKI (HRS-AKI versus non–HRS-AKI), with liver transplantation considered a competing risk.** In this analysis, liver transplantation was treated as a competing risk, and patients were censored from the model at the time they underwent the procedure.

In survival analysis, the HRS-AKI group exhibited an unadjusted trend toward a lower cumulative incidence of mortality than the non–HRS-AKI group (Figure 2). Nevertheless, in this multivariable analysis, using the non–HRS-AKI group as reference, the adjusted subdistribution hazard ratio (sHR) of HRS-AKI for 90-day mortality was 1.36 (95% confidence interval [CI], 0.95 to 1.94; *P* = 0.09). The clinical parameters independently associated with higher 90-day mortality included White race (sHR, 1.41; 95% CI, 1.01 to 1.96), exposure to vasopressors for shock (sHR, 1.62; 95% CI, 1.15 to 2.28), need for CRRT as initial RRT modality (sHR, 2.65; 95% CI, 1.87 to 3.77), and higher MELD-Na score (sHR, 1.04; 95% CI, 1.02 to 1.05). Being listed for liver transplant was associated with lower 90-day mortality (sHR, 0.19; 95% CI, 0.12 to 0.31) (Table 3).

**Table 3:** Univariable and multivariable Fine–Gray subdistribution hazard models for 90-day mortality in patients with cirrhosis and AKI requiring RRT

Parameter	Univariable Fine–Gray Model	*P* Value	Multivariable Fine–Gray Model	*P* Value	Multivariable Fine–Gray Model^a^	*P* Value
	sHR (95% CI)		sHR (95% CI)		sHR (95% CI)	
Etiology of AKI: HRS-AKI (versus non–HRS-AKI)	0.72 (0.50 to 1.04)	0.08	1.36 (0.95 to 1.94)	0.09	1.45 (0.99 to 2.12)	0.06
Age (per 1-yr)	1.00 (0.99 to 1.01)	0.80	1.01 (1.00 to 1.02)	0.15	1.01 (1.00 to 1.02)	0.15
Sex: female (versus male)	0.91 (0.70 to 1.18)	0.50	0.94 (0.71 to 1.25)	0.67	0.94 (0.71 to 1.25)	0.69
Race: White (versus non-White)	1.08 (0.80 to 1.46)	0.60	1.41 (1.01 to 1.96)	0.04	1.41 (1.02 to 1.96)	0.04
Ethnicity: Hispanic (versus non-Hispanic)	0.69 (0.44 to 1.08)	0.10	0.69 (0.44 to 1.06)	0.09	0.69 (0.45 to 1.07)	0.09
Volume capacity: nonhigh (versus high)	1.03 (0.78 to 1.37)	0.80	1.24 (0.91 to 1.69)	0.17	1.23 (0.91 to 1.68)	0.18
Vasopressor for shock: yes (versus no)	1.94 (1.42 to 2.65)	<0.001	1.62 (1.15 to 2.28)	0.006	1.63 (1.15 to 2.29)	0.006
Initial RRT modality: CRRT (versus IHD)	2.58 (1.86 to 3.57)	<0.001	2.65 (1.87 to 3.77)	<0.001	2.67 (1.88 to 3.79)	<0.001
MELD-Na score (per one-point)	1.02 (1.01 to 1.04)	0.004	1.04 (1.02 to 1.05)	<0.001	1.04 (1.02 to 1.05)	<0.001
Liver transplant: listed (versus nonlisted)	0.23 (0.15 to 0.34)	<0.001	0.19 (0.12 to 0.31)	<0.001	0.21 (0.12 to 0.36)	<0.001
Interaction: HRS-AKI^a^ liver transplant listed	—	—	—	—	0.76 (0.29 to 2.00)	0.58

CI, confidence interval; CRRT, continuous renal replacement therapy; HRS, hepatorenal syndrome; IHD, intermittent hemodialysis; RRT, renal replacement therapy; sHR, subdistribution hazard ratio.

a Interaction between hepatorenal syndrome-renal replacement therapy and liver transplant listed was evaluated in this model.

### Interaction Testing and Sensitivity Analyses

We did not find a significant interaction between study site RRT volume capacity and AKI etiology for 90-day mortality outcome (*P* interaction = 0.96). There was no significant interaction between liver transplant listing status and AKI etiology for 90-day mortality outcome (*P* interaction = 0.58), but the adjusted sHR increased after the inclusion of this interaction term in the model (sHR, 1.45; 95% CI, 0.99 to 2.12) (Table 3). There was no association between the AKI etiology and 90-day mortality when HRS-AKI was compared with only ATN as the reference (sHR, 1.14; 95% CI, 0.77 to 1.69) (Supplemental Table 5). When including CLIF-C ACLF scores in the main model, the sHR increased to 1.40 (95% CI, 0.99 to 1.98), and higher CLIF-C ACLF scores were found to be independently associated with higher 90-day mortality (sHR, 1.03; 95% CI, 1.02 to 1.05) (Supplemental Table 6).

## Discussion

In this study, we found that among hospitalized adult patients with cirrhosis and AKI-RRT, the overall mortality rate was high (59.6%) and rose further in those not listed for liver transplant (70.9%) or who initiated RRT with CRRT (68.9%). However, although elevated, these mortality rates are comparable with in-hospital mortality rates reported in general hospitalized patients with AKI-RRT (40%–60%)^34–38^ and in patients with AKI receiving CRRT (50%–70%)^38–40^ across various clinical settings. HRS-AKI was not independently associated with an increased risk of 90-day mortality when compared with other AKI etiologies (non–HRS-AKI or ATN). By contrast, White race, exposure to vasopressors for shock, the receipt of CRRT (versus IHD), higher MELD-Na, and higher CLIF-C ACLF score were independently associated with higher 90-day mortality, whereas being listed for liver transplant was associated with a lower risk of 90-day mortality.

Among the HRS-HARMONY cohort, the 90-day mortality rate was higher in patients with cirrhosis and AKI who received RRT (59.6%) versus those who did not (31.3%).^14^ This observation is consistent with previous studies and confirms that patients with cirrhosis and AKI-RRT have a poor prognosis.^11,18^ In previous studies, the reported mortality rates in these patients ranged between 60% and 90%, independent of the liver transplant listing status.^11,18,27,29^ This broad range may be explained by differences in study populations, processes of care, follow-up periods, and rates of liver transplantation. Allegretti *et al*. reported higher mortality (74.0%) in patients with cirrhosis and AKI-RRT than our study, but the follow-up period was 6 months (29), whereas Belcher *et al*. reported similar in-hospital mortality to our study (63.0%).^11^ In a European population, Staufer *et al*. reported a higher 3-month mortality (91.0%) in patients with cirrhosis and AKI-RRT, but the liver transplant rate was significantly lower than in our study (3.9% versus 17.1%).^18^

In patients with cirrhosis and AKI-RRT not listed for liver transplantation, Allegretti *et al*. reported a higher 6-month mortality than in those listed: 85% versus 46%, respectively. Similarly, Staufer *et al*. reported higher 3-month mortality in patients with cirrhosis and AKI-RRT not listed for liver transplantation (93% versus 86%), but no difference in mortality at 1 year (93% versus 91%). Importantly, the study by Allegretti *et al*. included a higher percentage of listed patients bridged for liver transplant than the report by Staufer *et al*. (48% versus 14%).^18,29^

We did not find significant mortality differences when comparing HRS-AKI with non–HRS-AKI, suggesting that the etiology of AKI may not be a major factor contributing to prognosis in this acutely ill population. Previous studies have reported differences in mortality when patients with cirrhosis and AKI were compared according to AKI etiology.^27,28^ However, these studies were not limited to patients receiving RRT but included a wide range of AKI severity. Similar to our multicenter study, a single-center study of 472 patients with cirrhosis limited to those with AKI-RRT did not find a difference in 6-month mortality when comparing HRS-AKI versus ATN.^29^ Furthermore, a single-center study by Saraiva *et al*. reported no differences in 3-month mortality when comparing 66 patients with cirrhosis and AKI-RRT because of only ATN versus HRS-AKI±ATN.^30^ Although our study was conducted (similar to the aforementioned studies) during a time in which terlipressin was not available for clinical use in the United States, these results, collectively, show that AKI-RRT is strongly associated with increased mortality in hospitalized patients with cirrhosis and this mortality is independent of AKI etiology.

As expected, acuity of illness parameters, including exposure to vasopressors for shock, MELD-Na score, and CLIF-C ACLF score, and not being listed for liver transplant were independently associated with increased mortality. These results are consistent with previous studies of patients with cirrhosis, both across all severity levels of AKI^27,28^ and in those including only patients with AKI-RRT.^25,29^ In our study, White race was independently associated with increased 90-day mortality. However, in previous studies, Black patients with cirrhosis had higher mortality and less access to lifesaving procedures, including liver transplantation.^41,42^ Importantly, studies have shown that once patients with cirrhosis are listed for transplant, clinical outcomes are comparable across race categories.^41,43^ The pathobiologic correlations of these observations are not fully understood, and additional studies are required to evaluate race, ethnicity, social determinants of health, and their interactions as factors influencing outcomes in hospitalized patients with cirrhosis and with or without AKI.^44^

Regarding the initial RRT modality, CRRT was more frequently used and was associated with higher mortality when compared with IHD. This is consistent with previous studies, which reported higher mortality in patients with cirrhosis who initiated with CRRT as compared with IHD.^11,25,26^ Such observational data do, however, carry a high risk of indication bias because patients treated with CRRT are more severely ill than their counterparts selected for IHD. In our study, despite adjusting the models by acuity of illness parameters, the possibility of unmeasured confounding remains.

The management of RRT in patients with cirrhosis and AKI is challenging. These patients are usually excluded from prospective studies because of the severity of their acute illness, and, when included, the etiology of AKI is seldom or insufficiently reported. Therefore, there is a lack of consensus regarding best RRT practices specific to hospitalized patients with cirrhosis and severe AKI.^1,2,45^ HRS-AKI is often considered to have a uniquely grim prognosis and is frequently cited as a deciding factor in decisions regarding RRT initiation. In patients listed for a liver transplant, RRT is recommended as a bridge therapy to transplantation.^2,21,46^ In severely ill patients with HRS-AKI not listed for liver transplant, however, RRT is usually avoided because of the risk of complications, such as intradialytic hypotension, bleeding, catheter-related complications, and, most importantly, the perceived futility of treatment. The increased use of effective vasoconstrictor therapy for HRS, including the newly US Food and Drug Administration–approved terlipressin, has allowed for the consideration of short duration (*i.e*., few days) RRT trials while awaiting response. However, controversy persists because RRT in patients with HRS-AKI has not demonstrated improved transplant-free survival or quality of life. In non–HRS-AKI patients not listed for liver transplant, RRT is recommended according to standard solute and fluid management indications and overall prognosis.^2,46–49^ However, the similar 90-day mortality rates for patients not listed for liver transplant with AKI-RRT across both HRS-AKI and non–HRS-AKI groups underscore that AKI etiology should not be a decisive factor when considering dialysis indication.

Although our study constitutes a large multicenter observational report with rigorously adjudicated liver cirrhosis, AKI, and AKI etiology data from 11 hospital network systems, we also acknowledge some limitations. First, this is a retrospective analysis, and our results, therefore, highlight important associations, but do not infer causality. Second, the duration of RRT and specific details of its prescription were not collected. A long duration of pretransplant RRT may increase the risk of mortality after transplantation.^26,50^ Third, the diagnosis of HRS-AKI is challenging, even for well-trained professionals. A clinical superimposition exists between the diagnosis of HRS-AKI and ATN, and diagnostically confounding factors are often present. However, in this cohort, each patient had two independent adjudicators who agreed on the etiology of AKI, with a third referee providing a tiebreaker diagnosis when necessary.

In this large contemporary multicenter cohort of hospitalized adult patients with cirrhosis and AKI-RRT, HRS-AKI was not independently associated with an increased risk of 90-day mortality when compared with other AKI etiologies, including ATN. In contrast to acuity of illness parameters, the etiology of AKI appears less relevant than previously considered when evaluating the prognosis of hospitalized adult patients with cirrhosis and AKI-RRT. Importantly, the overall mortality rate of this hospitalized adult population with cirrhosis and AKI-RRT was comparable with that reported for other hospitalized AKI-RRT populations. Future studies should focus on identifying best RRT practices, including nil exposure, to ameliorate outcomes for hospitalized patients with cirrhosis and severe AKI.

## Supplementary Material

## Data Availability

Partial restrictions to the data and/or materials apply. The datasets generated and analyzed during this study are available from the corresponding author on reasonable request.

## Supplemental Material

Supplemental Table 1. Self-reported volume capacity information of HRS-HARMONY consortium participating institutions during the study period.

Supplemental Table 2. Demographics and clinical characteristics of the study cohort by initial RRT modality.

Supplemental Table 3. Demographics and clinical characteristics of the study cohort by etiology of AKI.

Supplemental Table 4. Demographics and clinical characteristics of the study cohort by liver transplant listed status.

Supplemental Table 5. Multivariable Fine–Gray subdistribution hazard sensitivity analysis for 90-day mortality in patients with cirrhosis and AKI-RRT (HRS-AKI versus ATN).

Supplemental Table 6. Multivariable Fine–Gray subdistribution hazard sensitivity analysis for 90-day mortality in patients with cirrhosis and AKI-RRT, including CLIF-C ACLF score in the model.
