# Supplementary material for: Association of Hepatorenal Syndrome-Acute Kidney Injury with Mortality in Patients with Cirrhosis Requiring Renal Replacement Therapy: Results from the HRS-HARMONY Consortium
Source: Kidney360. 2024 Sep 30;6(2):247–56. doi: 10.34067/KID.0000000589 (PMC11882256; doi:10.34067/KID.0000000589)
Supplement: Supplementary file 1 [file kidney360-6-247-s001.pdf]

ASN American Society of Nephrology | JASN Journal of the American Society of Nephrology | KIDNEY360 Accessing Our World From Every Angle | CJASN Clinical Journal of the American Society of Nephrology

ASN Journal Disclosure Form

As per ASN journal policy, I have disclosed any financial relationships or commitments I have held in the past 36 months as included below. I have listed my Current Employer below to indicate there is a relationship requiring disclosure. If no relationship exists, my Current Employer is not listed.

A. Allegretti reports the following:
Employer: MGH; and Consultancy: Mallinckrodt Pharmaceuticals, Ocelot Bio, Motric Bio, Sequana Medical, Bioporto.

I understand that the information above will be published within the journal article, if accepted, and that failure to comply and/or to accurately and completely report the potential financial conflicts of interest could lead to the following: 1) Prior to publication, article rejection, or 2) Post-publication, sanctions ranging from, but not limited to, issuing a correction, reporting the inaccurate information to the authors' institution, banning authors from submitting work to ASN journals for varying lengths of time, and/or retraction of the published work.

Name: Andrew S. Allegretti
Manuscript ID: K360-2024-000415R1
Manuscript Title: Association of HRS-AKI with mortality in patients with cirrhosis requiring renal replacement therapy: Results from the HRS-HARMONY Consortium
Date of Completion: August 6, 2024
Disclosure Updated Date: July 13, 2024

ASN American Society of Nephrology JASN Journal of the American Society of Nephrology KIDNEY360 Accessing Our World From Every Angle CJASN Clinical Journal of the American Society of Nephrology

ASN Journal Disclosure Form

As per ASN journal policy, I have disclosed any financial relationships or commitments I have held in the past 36 months as included below. I have listed my Current Employer below to indicate there is a relationship requiring disclosure. If no relationship exists, my Current Employer is not listed.

J. Belcher reports the following:
Employer: Yale University; Consultancy: Mallinckrodt Pharmaceuticals; Honoraria: Mallinckrodt Pharmaceuticals; and Advisory or Leadership Role: Mallinckrodt Pharmaceuticals.

I understand that the information above will be published within the journal article, if accepted, and that failure to comply and/or to accurately and completely report the potential financial conflicts of interest could lead to the following: 1) Prior to publication, article rejection, or 2) Post-publication, sanctions ranging from, but not limited to, issuing a correction, reporting the inaccurate information to the authors' institution, banning authors from submitting work to ASN journals for varying lengths of time, and/or retraction of the published work.

Name: Justin Miles Belcher
Manuscript ID: K360-2024-000415R2
Manuscript Title: Association of HRS-AKI with mortality in patients with cirrhosis requiring renal replacement therapy: Results from the HRS-HARMONY Consortium
Date of Completion: September 4, 2024
Disclosure Updated Date: September 4, 2024

ASN American Society of Nephrology | JASN Journal of the American Society of Nephrology | KIDNEY360 Accessing Our World From Every Angle | CJASN Clinical Journal of the American Society of Nephrology

ASN Journal Disclosure Form

As per ASN journal policy, I have disclosed any financial relationships or commitments I have held in the past 36 months as included below. I have listed my Current Employer below to indicate there is a relationship requiring disclosure. If no relationship exists, my Current Employer is not listed.

A. Cama-Olivares reports the following:
Employer: University of Alabama at Birmingham; Brookwood Baptist Health

I understand that the information above will be published within the journal article, if accepted, and that failure to comply and/or to accurately and completely report the potential financial conflicts of interest could lead to the following: 1) Prior to publication, article rejection, or 2) Post-publication, sanctions ranging from, but not limited to, issuing a correction, reporting the inaccurate information to the authors' institution, banning authors from submitting work to ASN journals for varying lengths of time, and/or retraction of the published work.

Name: Augusto Cama-Olivares
Manuscript ID: K360-2024-000415R2
Manuscript Title: Association of HRS-AKI with mortality in patients with cirrhosis requiring renal replacement therapy: Results from the HRS-HARMONY Consortium
Date of Completion: September 3, 2024
Disclosure Updated Date: September 3, 2024

ASN American Society of Nephrology | JASN Journal of the American Society of Nephrology | KIDNEY360 Accessing Our World From Every Angle | CJASN Clinical Journal of the American Society of Nephrology

ASN Journal Disclosure Form

As per ASN journal policy, I have disclosed any financial relationships or commitments I have held in the past 36 months as included below. I have listed my Current Employer below to indicate there is a relationship requiring disclosure. If no relationship exists, my Current Employer is not listed.

R. Chung reports the following:
Employer: MGH; Research Funding: Abbvie, Janssen, Boehringer Ingelheim, BMS, GSK, Salix (all to institution); and Patents or Royalties: Ferrumax.

I understand that the information above will be published within the journal article, if accepted, and that failure to comply and/or to accurately and completely report the potential financial conflicts of interest could lead to the following: 1) Prior to publication, article rejection, or 2) Post-publication, sanctions ranging from, but not limited to, issuing a correction, reporting the inaccurate information to the authors' institution, banning authors from submitting work to ASN journals for varying lengths of time, and/or retraction of the published work.

Name: Raymond T. Chung
Manuscript ID: K360-2024-000415R1
Manuscript Title: ssociation of HRS-AKI with mortality in patients with cirrhosis requiring renal replacement therapy: Results from the HRS-HARMONY Consortium,
Date of Completion: August 6, 2024
Disclosure Updated Date: August 6, 2024

ASN
American Society of Nephrology
JASN
Journal of the American Society of Nephrology
KIDNEY360
Accessing Our World From Every Angle
CJASN
Clinical Journal of the American Society of Nephrology

ASN Journal Disclosure Form

As per ASN journal policy, I have disclosed any financial relationships or commitments I have held in the past 36 months as included below. I have listed my Current Employer below to indicate there is a relationship requiring disclosure. If no relationship exists, my Current Employer is not listed.

G. Cullaro reports the following:
Employer: Columbia University; Consultancy: Ocelot Bio; Retro; and Honoraria: Ocelot Bio.

I understand that the information above will be published within the journal article, if accepted, and that failure to comply and/or to accurately and completely report the potential financial conflicts of interest could lead to the following: 1) Prior to publication, article rejection, or 2) Post-publication, sanctions ranging from, but not limited to, issuing a correction, reporting the inaccurate information to the authors' institution, banning authors from submitting work to ASN journals for varying lengths of time, and/or retraction of the published work.

Name: Giuseppe Cullaro
Manuscript ID: K360-2024-000415R2
Manuscript Title: Association of HRS-AKI with mortality in patients with cirrhosis requiring renal replacement therapy: Results from the HRS-HARMONY Consortium
Date of Completion: September 16, 2024
Disclosure Updated Date: September 16, 2024

ASN American Society of Nephrology | JASN Journal of the American Society of Nephrology | KIDNEY360 Accessing Our World From Every Angle | CJASN Clinical Journal of the American Society of Nephrology

ASN Journal Disclosure Form

As per ASN journal policy, I have disclosed any financial relationships or commitments I have held in the past 36 months as included below. I have listed my Current Employer below to indicate there is a relationship requiring disclosure. If no relationship exists, my Current Employer is not listed.

C. Karvellas reports the following:
Employer: University of Alberta; and Honoraria: Baxter.

I understand that the information above will be published within the journal article, if accepted, and that failure to comply and/or to accurately and completely report the potential financial conflicts of interest could lead to the following: 1) Prior to publication, article rejection, or 2) Post-publication, sanctions ranging from, but not limited to, issuing a correction, reporting the inaccurate information to the authors' institution, banning authors from submitting work to ASN journals for varying lengths of time, and/or retraction of the published work.

Name: Constantine Karvellas
Manuscript ID: K360-2024-000415R2
Manuscript Title: Association of HRS-AKI with mortality in patients with cirrhosis requiring renal replacement therapy: Results from the HRS-HARMONY Consortium
Date of Completion: September 3, 2024
Disclosure Updated Date: September 3, 2024

ASN American Society of Nephrology | JASN Journal of the American Society of Nephrology | KIDNEY360 Accessing Our World From Every Angle | CJASN Clinical Journal of the American Society of Nephrology

ASN Journal Disclosure Form

As per ASN journal policy, I have disclosed any financial relationships or commitments I have held in the past 36 months as included below. I have listed my Current Employer below to indicate there is a relationship requiring disclosure. If no relationship exists, my Current Employer is not listed.

J. Levitsky reports the following:
Employer: Northwestern; Consultancy: Eurofins; Mallinckrodt; Research Funding: Eurofins;; Honoraria: Mallinckrodt; Patents or Royalties: Eurofins; and Speakers Bureau: Mallinckrodt; Takeda.

I understand that the information above will be published within the journal article, if accepted, and that failure to comply and/or to accurately and completely report the potential financial conflicts of interest could lead to the following: 1) Prior to publication, article rejection, or 2) Post-publication, sanctions ranging from, but not limited to, issuing a correction, reporting the inaccurate information to the authors' institution, banning authors from submitting work to ASN journals for varying lengths of time, and/or retraction of the published work.

Name: Josh Levitsky
Manuscript ID: K360-2024-000415R2
Manuscript Title: Association of HRS-AKI with mortality in patients with cirrhosis requiring renal replacement therapy: Results from the HRS-HARMONY Consortium
Date of Completion: September 5, 2024
Disclosure Updated Date: May 21, 2024

ASN American Society of Nephrology | JASN Journal of the American Society of Nephrology | KIDNEY360 Accessing Our World From Every Angle | CJASN Clinical Journal of the American Society of Nephrology

ASN Journal Disclosure Form

As per ASN journal policy, I have disclosed any financial relationships or commitments I have held in the past 36 months as included below. I have listed my Current Employer below to indicate there is a relationship requiring disclosure. If no relationship exists, my Current Employer is not listed.

J. Neyra reports the following:
Employer: University of Alabama at Birmingham; Consultancy: Baxter, AcelRx, Spectral Medical Inc.; and Advisory or Leadership Role: Section Editor, Clinical Nephrology; Guest Editor, Critical Care Nephrology in Advances in Kidney Disease and Health; Editorial Board, Kidney360, American Journal of Kidney Diseases, Advances in Kidney Disease and Health; Co-Director, Clinical & Innovation Cores of the UAB-UCSD O'Brien Center for Acute Kidney Injury Research.

I understand that the information above will be published within the journal article, if accepted, and that failure to comply and/or to accurately and completely report the potential financial conflicts of interest could lead to the following: 1) Prior to publication, article rejection, or 2) Post-publication, sanctions ranging from, but not limited to, issuing a correction, reporting the inaccurate information to the authors' institution, banning authors from submitting work to ASN journals for varying lengths of time, and/or retraction of the published work.

Name: Javier A. Neyra
Manuscript ID: K360-2024-000415R2
Manuscript Title: Association of HRS-AKI with mortality in patients with cirrhosis requiring renal replacement therapy: Results from the HRS-HARMONY Consortium
Date of Completion: September 3, 2024
Disclosure Updated Date: May 10, 2024

ASN Journal Disclosure Form

As per ASN journal policy, I have disclosed any financial relationships or commitments I have held in the past 36 months as included below. I have listed my Current Employer below to indicate there is a relationship requiring disclosure. If no relationship exists, my Current Employer is not listed.

T. Ouyang reports the following:
Employer: Massachusetts General Hospital

I understand that the information above will be published within the journal article, if accepted, and that failure to comply and/or to accurately and completely report the potential financial conflicts of interest could lead to the following: 1) Prior to publication, article rejection, or 2) Post-publication, sanctions ranging from, but not limited to, issuing a correction, reporting the inaccurate information to the authors' institution, banning authors from submitting work to ASN journals for varying lengths of time, and/or retraction of the published work.

Name: Tianqi Ouyang
Manuscript ID: K360-2024-000415R2
Manuscript Title: Association of HRS-AKI with mortality in patients with cirrhosis requiring renal replacement therapy: Results from the HRS-HARMONY Consortium
Date of Completion: September 13, 2024
Disclosure Updated Date: May 16, 2024

ASN
American Society of Nephrology
JASN
Journal of the American Society of Nephrology
KIDNEY360
Accessing Our World From Every Angle
CJASN
Clinical Journal of the American Society of Nephrology

ASN Journal Disclosure Form

As per ASN journal policy, I have disclosed any financial relationships or commitments I have held in the past 36 months as included below. I have listed my Current Employer below to indicate there is a relationship requiring disclosure. If no relationship exists, my Current Employer is not listed.

E. Orman reports the following:
Employer: Indiana University School of Medicine; Consultancy: Sitero; and Advisory or Leadership Role: Biovie.

I understand that the information above will be published within the journal article, if accepted, and that failure to comply and/or to accurately and completely report the potential financial conflicts of interest could lead to the following: 1) Prior to publication, article rejection, or 2) Post-publication, sanctions ranging from, but not limited to, issuing a correction, reporting the inaccurate information to the authors' institution, banning authors from submitting work to ASN journals for varying lengths of time, and/or retraction of the published work.

Name: Eric S. Orman
Manuscript ID: K360-2024-000415R1
Manuscript Title: Association of HRS-AKI with mortality in patients with cirrhosis requiring renal replacement therapy: Results from the HRS-HARMONY Consortium
Date of Completion: August 6, 2024
Disclosure Updated Date: August 6, 2024

ASN American Society of Nephrology
JASN Journal of the American Society of Nephrology
KIDNEY360 Accessing Our World From Every Angle
CJASN Clinical Journal of the American Society of Nephrology

ASN Journal Disclosure Form

As per ASN journal policy, I have disclosed any financial relationships or commitments I have held in the past 36 months as included below. I have listed my Current Employer below to indicate there is a relationship requiring disclosure. If no relationship exists, my Current Employer is not listed.

K. Patidar reports the following:
Employer: Baylor College of Medicine; and Advisory or Leadership Role: Madrigal Pharmaceuticals.

I understand that the information above will be published within the journal article, if accepted, and that failure to comply and/or to accurately and completely report the potential financial conflicts of interest could lead to the following: 1) Prior to publication, article rejection, or 2) Post-publication, sanctions ranging from, but not limited to, issuing a correction, reporting the inaccurate information to the authors' institution, banning authors from submitting work to ASN journals for varying lengths of time, and/or retraction of the published work.

Name: Kavish Rohit Patidar
Manuscript ID: K360-2024-000415R2
Manuscript Title: Association of HRS-AKI with mortality in patients with cirrhosis requiring renal replacement therapy: Results from the HRS-HARMONY Consortium
Date of Completion: September 5, 2024
Disclosure Updated Date: August 6, 2024

ASN American Society of Nephrology | JASN Journal of the American Society of Nephrology | KIDNEY360 Accessing Our World From Every Angle | CJASN Clinical Journal of the American Society of Nephrology

ASN Journal Disclosure Form

As per ASN journal policy, I have disclosed any financial relationships or commitments I have held in the past 36 months as included below. I have listed my Current Employer below to indicate there is a relationship requiring disclosure. If no relationship exists, my Current Employer is not listed.

K. Regner reports the following:
Employer: Medical College of Wisconsin; Consultancy: Mallinckrodt Pharma; Honoraria: Mallinckrodt; and Speakers Bureau: Mallinckrodt Pharma.

I understand that the information above will be published within the journal article, if accepted, and that failure to comply and/or to accurately and completely report the potential financial conflicts of interest could lead to the following: 1) Prior to publication, article rejection, or 2) Post-publication, sanctions ranging from, but not limited to, issuing a correction, reporting the inaccurate information to the authors' institution, banning authors from submitting work to ASN journals for varying lengths of time, and/or retraction of the published work.

Name: Kevin R. Regner
Manuscript ID: K360-2024-000415R1
Manuscript Title: Association of HRS-AKI with mortality in patients with cirrhosis requiring renal replacement therapy: Results from the HRS-HARMONY Consortium
Date of Completion: August 6, 2024
Disclosure Updated Date: August 6, 2024

ASN American Society of Nephrology | JASN Journal of the American Society of Nephrology | KIDNEY360 Accessing Our World From Every Angle | CJASN Clinical Journal of the American Society of Nephrology

ASN Journal Disclosure Form

As per ASN journal policy, I have disclosed any financial relationships or commitments I have held in the past 36 months as included below. I have listed my Current Employer below to indicate there is a relationship requiring disclosure. If no relationship exists, my Current Employer is not listed.

J. Robinson reports the following:
Employer: Massachusetts General Hospital; and Other Interests or Relationships: Massachusetts General Hospital.

I understand that the information above will be published within the journal article, if accepted, and that failure to comply and/or to accurately and completely report the potential financial conflicts of interest could lead to the following: 1) Prior to publication, article rejection, or 2) Post-publication, sanctions ranging from, but not limited to, issuing a correction, reporting the inaccurate information to the authors' institution, banning authors from submitting work to ASN journals for varying lengths of time, and/or retraction of the published work.

Name: Jevon E. Robinson
Manuscript ID: K360-2024-000415R1
Manuscript Title: Association of HRS-AKI with mortality in patients with cirrhosis requiring renal replacement therapy: Results from the HRS-HARMONY Consortium
Date of Completion: August 6, 2024
Disclosure Updated Date: August 6, 2024

ASN American Society of Nephrology JASN Journal of the American Society of Nephrology KIDNEY360 Accessing Our World From Every Angle CJASN Clinical Journal of the American Society of Nephrology

ASN Journal Disclosure Form

As per ASN journal policy, I have disclosed any financial relationships or commitments I have held in the past 36 months as included below. I have listed my Current Employer below to indicate there is a relationship requiring disclosure. If no relationship exists, my Current Employer is not listed.

D. Saly has nothing to disclose.

I understand that the information above will be published within the journal article, if accepted, and that failure to comply and/or to accurately and completely report the potential financial conflicts of interest could lead to the following: 1) Prior to publication, article rejection, or 2) Post-publication, sanctions ranging from, but not limited to, issuing a correction, reporting the inaccurate information to the authors' institution, banning authors from submitting work to ASN journals for varying lengths of time, and/or retraction of the published work.

Name: Danielle L. Saly
Manuscript ID: K360-2024-000415R1
Manuscript Title: Association of HRS-AKI with mortality in patients with cirrhosis requiring renal replacement therapy: Results from the HRS-HARMONY Consortium
Date of Completion: August 9, 2024
Disclosure Updated Date: April 4, 2024

ASN American Society of Nephrology | JASN Journal of the American Society of Nephrology | KIDNEY360 Accessing Our World From Every Angle | CJASN Clinical Journal of the American Society of Nephrology

ASN Journal Disclosure Form

As per ASN journal policy, I have disclosed any financial relationships or commitments I have held in the past 36 months as included below. I have listed my Current Employer below to indicate there is a relationship requiring disclosure. If no relationship exists, my Current Employer is not listed.

D. Sawinski reports the following:
Employer: Weill Cornell Medical College; Ownership Interest: CareDx; Research Funding: National Institutes of Health; Patents or Royalties: Uptodate; Advisory or Leadership Role: American Journal of Kidney Diseases; Clinical Transplantation; Councilor at Large, American Society of Transplantation Board of Directors; and Other Interests or Relationships: UNOS MPSC Committee member; Expert witness testimony.

I understand that the information above will be published within the journal article, if accepted, and that failure to comply and/or to accurately and completely report the potential financial conflicts of interest could lead to the following: 1) Prior to publication, article rejection, or 2) Post-publication, sanctions ranging from, but not limited to, issuing a correction, reporting the inaccurate information to the authors' institution, banning authors from submitting work to ASN journals for varying lengths of time, and/or retraction of the published work.

Name: Deirdre L. Sawinski
Manuscript ID: K360-2024-000415R1
Manuscript Title: Association of HRS-AKI with mortality in patients with cirrhosis requiring renal replacement therapy: Results from the HRS-HARMONY Consortium
Date of Completion: August 6, 2024
Disclosure Updated Date: August 6, 2024

ASN American Society of Nephrology JASN Journal of the American Society of Nephrology KIDNEY360 Accessing Our World From Every Angle CJASN Clinical Journal of the American Society of Nephrology

ASN Journal Disclosure Form

As per ASN journal policy, I have disclosed any financial relationships or commitments I have held in the past 36 months as included below. I have listed my Current Employer below to indicate there is a relationship requiring disclosure. If no relationship exists, my Current Employer is not listed.

P. Sharma reports the following:
Employer: Michigan Medicine, University of Michigan; and Advisory or Leadership Role: Associate Editor Liver Transplantation Journal.

I understand that the information above will be published within the journal article, if accepted, and that failure to comply and/or to accurately and completely report the potential financial conflicts of interest could lead to the following: 1) Prior to publication, article rejection, or 2) Post-publication, sanctions ranging from, but not limited to, issuing a correction, reporting the inaccurate information to the authors' institution, banning authors from submitting work to ASN journals for varying lengths of time, and/or retraction of the published work.

Name: Pratima Sharma
Manuscript ID: K360-2024-000415R1
Manuscript Title: Association of HRS-AKI with mortality in patients with cirrhosis requiring renal replacement therapy: Results from the HRS-HARMONY Consortium
Date of Completion: August 6, 2024
Disclosure Updated Date: August 6, 2024

ASN American Society of Nephrology
JASN Journal of the American Society of Nephrology
KIDNEY360 Accessing Our World From Every Angle
CJASN Clinical Journal of the American Society of Nephrology

ASN Journal Disclosure Form

As per ASN journal policy, I have disclosed any financial relationships or commitments I have held in the past 36 months as included below. I have listed my Current Employer below to indicate there is a relationship requiring disclosure. If no relationship exists, my Current Employer is not listed.

S. St. Hillien reports the following:
Employer: Mass General Hospital

I understand that the information above will be published within the journal article, if accepted, and that failure to comply and/or to accurately and completely report the potential financial conflicts of interest could lead to the following: 1) Prior to publication, article rejection, or 2) Post-publication, sanctions ranging from, but not limited to, issuing a correction, reporting the inaccurate information to the authors' institution, banning authors from submitting work to ASN journals for varying lengths of time, and/or retraction of the published work.

Name: Shelsea A. St. Hillien
Manuscript ID: K360-2024-000415R2
Manuscript Title: Association of HRS-AKI with mortality in patients with cirrhosis requiring renal replacement therapy: Results from the HRS-HARMONY Consortium
Date of Completion: September 13, 2024
Disclosure Updated Date: September 13, 2024

ASN American Society of Nephrology · JASN Journal of the American Society of Nephrology · KIDNEY360 Accessing Our World From Every Angle · CJASN Clinical Journal of the American Society of Nephrology

ASN Journal Disclosure Form

As per ASN journal policy, I have disclosed any financial relationships or commitments I have held in the past 36 months as included below. I have listed my Current Employer below to indicate there is a relationship requiring disclosure. If no relationship exists, my Current Employer is not listed.

T. Takeuchi reports the following:
Employer: University of Alabama at Birmingham

I understand that the information above will be published within the journal article, if accepted, and that failure to comply and/or to accurately and completely report the potential financial conflicts of interest could lead to the following: 1) Prior to publication, article rejection, or 2) Post-publication, sanctions ranging from, but not limited to, issuing a correction, reporting the inaccurate information to the authors' institution, banning authors from submitting work to ASN journals for varying lengths of time, and/or retraction of the published work.

Name: Tomonori Takeuchi
Manuscript ID: K360-2024-000415R2
Manuscript Title: Association of HRS-AKI with mortality in patients with cirrhosis requiring renal replacement therapy: Results from the HRS-HARMONY Consortium
Date of Completion: September 3, 2024
Disclosure Updated Date: September 3, 2024

ASN American Society of Nephrology | JASN Journal of the American Society of Nephrology | KIDNEY360 Accessing Our World From Every Angle | CJASN Clinical Journal of the American Society of Nephrology

ASN Journal Disclosure Form

As per ASN journal policy, I have disclosed any financial relationships or commitments I have held in the past 36 months as included below. I have listed my Current Employer below to indicate there is a relationship requiring disclosure. If no relationship exists, my Current Employer is not listed.

J. Teixeira reports the following:
Consultancy: Outset Medical; Ownership Interest: Eli Lilly and Company, Novo Nordisk A/S, Pfizer Inc.; Research Funding: Sentien Biotechnologies Inc.; Rediscovery Life Sciences LLC; Gilead; La Jolla Pharmaceutical Company/Innoviva; Astute Medical/bioMérieux; Honoraria: Outset Medical; and Speakers Bureau: Outset Medical.

I understand that the information above will be published within the journal article, if accepted, and that failure to comply and/or to accurately and completely report the potential financial conflicts of interest could lead to the following: 1) Prior to publication, article rejection, or 2) Post-publication, sanctions ranging from, but not limited to, issuing a correction, reporting the inaccurate information to the authors' institution, banning authors from submitting work to ASN journals for varying lengths of time, and/or retraction of the published work.

Name: J. Pedro Teixeira
Manuscript ID: K360-2024-000415R1
Manuscript Title: Association of HRS-AKI with mortality in patients with cirrhosis requiring renal replacement therapy: Results from the HRS-HARMONY Consortium
Date of Completion: August 6, 2024
Disclosure Updated Date: August 6, 2024

ASN
American Society of Nephrology
JASN
Journal of the American Society of Nephrology
KIDNEY360
Accessing Our World From Every Angle
CJASN
Clinical Journal of the American Society of Nephrology

ASN Journal Disclosure Form

As per ASN journal policy, I have disclosed any financial relationships or commitments I have held in the past 36 months as included below. I have listed my Current Employer below to indicate there is a relationship requiring disclosure. If no relationship exists, my Current Employer is not listed.

N. Ufere reports the following:
Employer: MASSACHUSETTS GENERAL HOSPITAL

I understand that the information above will be published within the journal article, if accepted, and that failure to comply and/or to accurately and completely report the potential financial conflicts of interest could lead to the following: 1) Prior to publication, article rejection, or 2) Post-publication, sanctions ranging from, but not limited to, issuing a correction, reporting the inaccurate information to the authors' institution, banning authors from submitting work to ASN journals for varying lengths of time, and/or retraction of the published work.

Name: Nneka Ufere
Manuscript ID: K360-2024-000415R1
Manuscript Title: Association of HRS-AKI with mortality in patients with cirrhosis requiring renal replacement therapy: Results from the HRS-HARMONY Consortium
Date of Completion: August 6, 2024
Disclosure Updated Date: January 9, 2024

ASN American Society of Nephrology | JASN Journal of the American Society of Nephrology | KIDNEY360 Accessing Our World From Every Angle | CJASN Clinical Journal of the American Society of Nephrology

ASN Journal Disclosure Form

As per ASN journal policy, I have disclosed any financial relationships or commitments I have held in the past 36 months as included below. I have listed my Current Employer below to indicate there is a relationship requiring disclosure. If no relationship exists, my Current Employer is not listed.

J. Velez reports the following:
Employer: Ochsner Health; Consultancy: Mallinckrodt Pharmaceuticals, Travere Therapeutics, Calliditas; Honoraria: Mallinckrodt Pharmaceuticals, Travere Therapeutics, Calliditas; Advisory or Leadership Role: Mallinckrodt Pharmaceuticals, Travere Therapeutics, Calliditas; and Speakers Bureau: Mallinckrodt Pharmaceuticals.

I understand that the information above will be published within the journal article, if accepted, and that failure to comply and/or to accurately and completely report the potential financial conflicts of interest could lead to the following: 1) Prior to publication, article rejection, or 2) Post-publication, sanctions ranging from, but not limited to, issuing a correction, reporting the inaccurate information to the authors' institution, banning authors from submitting work to ASN journals for varying lengths of time, and/or retraction of the published work.

Name: Juan Carlos Q. Velez
Manuscript ID: K360-2024-000415R2
Manuscript Title: Association of HRS-AKI with mortality in patients with cirrhosis requiring renal replacement therapy: Results from the HRS-HARMONY Consortium
Date of Completion: September 16, 2024
Disclosure Updated Date: March 27, 2024

ASN Journal Disclosure Form

As per ASN journal policy, I have disclosed any financial relationships or commitments I have held in the past 36 months as included below. I have listed my Current Employer below to indicate there is a relationship requiring disclosure. If no relationship exists, my Current Employer is not listed.

H. Wadei reports the following:
Employer: Mayo Clinic; and Ownership Interest: Novo Nordisk; Elli Lly; Apple Inc.

I understand that the information above will be published within the journal article, if accepted, and that failure to comply and/or to accurately and completely report the potential financial conflicts of interest could lead to the following: 1) Prior to publication, article rejection, or 2) Post-publication, sanctions ranging from, but not limited to, issuing a correction, reporting the inaccurate information to the authors' institution, banning authors from submitting work to ASN journals for varying lengths of time, and/or retraction of the published work.

Name: Hani Wadei
Manuscript ID: K360-2024-000415R1
Manuscript Title: Association of HRS-AKI with mortality in patients with cirrhosis requiring renal replacement therapy: Results from the HRS-HARMONY Consortium
Date of Completion: August 6, 2024
Disclosure Updated Date: August 6, 2024

ASN American Society of Nephrology | JASN Journal of the American Society of Nephrology | KIDNEY360 Accessing Our World From Every Angle | CJASN Clinical Journal of the American Society of Nephrology

ASN Journal Disclosure Form

As per ASN journal policy, I have disclosed any financial relationships or commitments I have held in the past 36 months as included below. I have listed my Current Employer below to indicate there is a relationship requiring disclosure. If no relationship exists, my Current Employer is not listed.

N. Wahid has nothing to disclose.

I understand that the information above will be published within the journal article, if accepted, and that failure to comply and/or to accurately and completely report the potential financial conflicts of interest could lead to the following: 1) Prior to publication, article rejection, or 2) Post-publication, sanctions ranging from, but not limited to, issuing a correction, reporting the inaccurate information to the authors' institution, banning authors from submitting work to ASN journals for varying lengths of time, and/or retraction of the published work.

Name: Nabeel Wahid
Manuscript ID: K360-2024-000415R2
Manuscript Title: Association of HRS-AKI with mortality in patients with cirrhosis requiring renal replacement therapy: Results from the HRS-HARMONY Consortium
Date of Completion: September 5, 2024
Disclosure Updated Date: September 5, 2024
