# Supplementary material for: Association of Hepatorenal Syndrome-Acute Kidney Injury with Mortality in Patients with Cirrhosis Requiring Renal Replacement Therapy: Results from the HRS-HARMONY Consortium
Source: Kidney360. 2024 Sep 30;6(2):247–56. doi: 10.34067/KID.0000000589 (PMC11882256; doi:10.34067/KID.0000000589)
Supplement: Supplementary file 2 [file kidney360-6-247-s002.pdf]

SUPPLEMENTARY MATERIAL

Table. S1: Self-reported volume capacity information of HRS-HARMONY consortium participating institutions during the study period

Table. S2: Demographics and clinical characteristics of the study cohort by initial RRT modality

Table. S3: Demographics and clinical characteristics of the study cohort by etiology of acute kidney injury

Table. S4: Demographics and clinical characteristics of the study cohort by liver transplant listed status

Table. S5: Multivariable Fine-Gray subdistribution hazard sensitivity analysis for 90-day mortality in patients with cirrhosis and AKI-RRT (HRS-AKI vs. ATN)

Table. S6: Multivariable Fine-Gray subdistribution hazard sensitivity analysis for 90-day mortality in patients with cirrhosis and AKI-RRT, including CLIF-C ACLF score in the model

Supplementary Table S1: Self-reported volume capacity information of HRS-HARMONY consortium participating institutions during the study period

Participating institutions	Location	RRT volume capacity	Total number of ICU beds	Total number of HD devices	Total number of CRRT machines	Liver transplants per year	Kidney transplants per year
Mass General Brigham	Boston, MA	High	50-150	>20	>20	50 - 150	>150
Mayo Clinic	Rochester, MN	High	>150	>20	>20	50 - 150	>150
Indiana University Health	Indianapolis, IN	High	50-150	>20	>20	>150	>150
Baylor University Medical Center	Dallas, TX	Non-High	50-150	10 - 20	10 - 20	50 - 150	>150
University of Kentucky Hospital	Lexington, KY	Non-High	>150	10 - 20	>20	50 - 150	50 - 150
Yale New Haven Hospital	New Haven, CT	High	>150	>20	>20	<50	50 - 150
University of Michigan Hospital	Ann Arbor, MI	Non-High	50-150	10 - 20	>20	50 - 150	>150
Ochsner Medical Center	Jefferson, LA	Non-High	50-150	>20	10 - 20	>150	>150
Medical College of Wisconsin	Milwaukee, WI	High	50-150	>20	>20	50 - 150	50 - 150
Mayo Clinic	Jacksonville, FL	High	50-150	>20	>20	>150	>150
Keck Medical Center of the University of Southern California	Los Angeles, CA	Non-High	50-150	10 - 20	>20	50 - 150	>150

Abbreviations: ICU, intensive care unit; HD, hemodialysis; CRRT, continuous renal replacement therapy.

Supplementary Table S2: Demographics and clinical characteristics of the study cohort by initial RRT modality

Parameter	Initial RRT modality		p
	IHD (n = 108)	CRRT (n = 264)	
**Demographics**			
Age (years)	58 [48, 67]	59 [48, 65]	0.42
Female sex (%)	48 (44.4)	110 (41.7)	0.71
White race (%)	88 (81.5)	183 (69.3)	0.02
Hispanic ethnicity (%)	11 (10.2)	32 (12.1)	0.73
**Comorbidities**			
Etiology of cirrhosis (%)			0.46
Alcohol	40 (37.0)	115 (43.6)	
Hepatitis C	9 (8.3)	26 (9.8)	
MASLD	27 (25.0)	45 (17.0)	
Multifactorial	7 (6.5)	20 (7.6)	
Other	25 (23.2)	58 (22.0)	
Etiology of AKI (%)			0.04
Prerenal AKI	6 (5.6)	18 (6.8)	
HRS-AKI	28 (25.9)	37 (14.0)	
Acute tubular necrosis	58 (53.7)	175 (66.3)	
Other AKI	4 (3.7)	4 (1.5)	
Unable to classify	12 (11.1)	30 (11.4)	
Diabetes (%)	42 (38.9)	88 (33.3)	0.37
Coronary artery disease (%)	20 (18.5)	38 (14.4)	0.40
Chronic kidney disease (%)	45 (41.7)	45 (17.1)	<0.001
Hypertension (%)	61 (56.5)	109 (41.3)	0.01
**Complications of cirrhosis**			
Ascites (%)	91 (84.3)	224 (84.8)	0.99
Encephalopathy (%)	65 (60.7)	186 (70.5)	0.09
Gastrointestinal bleeding (%)	30 (28.0)	115 (43.6)	0.008
Spontaneous bacterial peritonitis (%)	13 (12.1)	63 (23.9)	0.02
Hepatocellular carcinoma (%)	5 (4.7)	18 (6.8)	0.59
**Characteristics of admission**			
ICU admission (%)	67 (62.0)	263 (99.6)	<0.001
Admission MAP (mmHg)	68.3 [60.9, 75.0]	64.5 [56.3, 73.3]	0.02
Vasopressor for shock (%)	49 (46.2)	208 (78.8)	<0.001
Vasoconstrictor for HRS (%)	68 (63.0)	192 (73.0)	0.07
Albumin given during admission (%)	89 (83.2)	229 (87.4)	0.37
Total albumin given during admission* (g)	100 [59, 175]	125 [75, 200]	0.07
Mechanical ventilation (%)	31 (28.7)	199 (75.7)	<0.001
Days from admission to RRT initiation†	4.0 [2.0, 10.5]	3.5 [1.0, 9.0]	0.08
**Survival Scores**			
MELD-Na score	30 [23, 34]	32 [26, 37]	0.04
CLIF-C ACLF score	53.8 [47.0, 59.5]	56.9 [50.5, 62.9]	0.02
**Laboratory values**			
Sodium (mEq/L)	134 [129, 138]	133 [128, 137]	0.68
Admission creatinine (mg/dL)	3.1 [2.0, 4.4]	2.3 [1.4, 3.6]	<0.001
Peak creatinine (mg/dL)	5.6 [4.0, 7.1]	3.6 [2.7, 5.0]	<0.001
BUN (mg/dL)	58 [33, 81]	41 [24, 60]	<0.001
White blood count (K/uL)	9.7 [6.2, 16.0]	10.3 [6.9, 16.0]	0.70
Albumin (g/dL)	3.0 [2.5, 3.4]	2.8 [2.4, 3.2]	0.03
International normalized ratio (INR)	1.6 [1.3, 2.2]	2.0 [1.6, 2.8]	<0.001
Total bilirubin (mg/dL)	3.6 [1.1, 11.7]	6.5 [2.9, 18.2]	0.001
FENa‡ (%)	0.4 [0.2, 0.8]	0.3 [0.2, 0.7]	0.34

Continuous variables are reported as median [interquartile range].

* Available in 270 patients.

† Available in 331 patients.

‡ Available in 228 patients.

Abbreviations: RRT, renal replacement therapy; IHD, intermittent hemodialysis; CRRT, continuous RRT; MASLD, Metabolic dysfunction-associated steatotic liver disease; AKI, acute kidney injury; HRS, hepatorenal syndrome; ICU, intensive care unit; MAP, mean arterial pressure; BUN, blood urea nitrogen; FENa, fractional excretion of sodium.

Supplementary Table S3: Demographics and clinical characteristics of the study cohort by etiology of acute kidney injury

Parameter	All Patients (n= 374)	Pre-renal AKI (n = 25)	Hepatorenal Syndrome (n = 65)	Acute Tubular Necrosis (n = 234)	Other AKI (n = 8)	Unable to Classify (n = 42)	p
**Demographics**							
Age (years)	58 [48, 65]	61 [48, 68]	58 [48, 64]	58 [46, 66]	55 [45, 59]	59 [52, 64]	0.61
Female sex (%)	160 (42.8)	16 (64.0)	31 (47.7)	92 (39.3)	4 (50.0)	17 (40.5)	0.16
White race (%)	271 (72.5)	16 (64.0)	50 (76.9)	177 (75.6)	4 (50.0)	24 (57.1)	0.05
Hispanic ethnicity (%)	43 (11.5)	7 (28.0)	11 (16.9)	15 (6.4)	2 (25.0)	8 (19.0)	0.001
**Comorbidities**							
Etiology of cirrhosis (%)							0.06
Alcohol	156 (41.7)	7 (28.0)	27 (41.5)	103 (44.0)	2 (25.0)	17 (40.5)	
Hepatitis C	35 (9.4)	5 (20.0)	5 (7.7)	21 (9.0)	1 (12.5)	3 (7.1)	
MASLD	72 (19.3)	5 (20.0)	23 (35.4)	32 (13.7)	1 (12.5)	11 (26.2)	
Multifactorial	28 (7.5)	3 (12.0)	1 (1.5)	21 (9.0)	1 (12.5)	2 (4.8)	
Other	83 (22.1)	5 (20.0)	9 (13.9)	57 (24.3)	3 (37.5)	9 (21.4)	
Diabetes (%)	130 (34.8)	9 (36.0)	23 (35.4)	78 (33.3)	4 (50.0)	16 (38.1)	0.87
Coronary artery disease (%)	58 (15.5)	3 (12.0)	7 (10.8)	37 (15.8)	1 (12.5)	10 (23.8)	0.46
Chronic kidney disease (%)	90 (24.1)	7 (28.0)	22 (34.4)	48 (20.5)	4 (50.0)	9 (21.4)	0.07
Hypertension (%)	170 (45.5)	12 (48.0)	27 (41.5)	102 (43.6)	7 (87.5)	22 (52.4)	0.12
**Complications of cirrhosis**							
Ascites (%)	317 (84.8)	15 (60.0)	63 (96.9)	197 (84.2)	3 (37.5)	39 (92.9)	<0.001
Encephalopathy (%)	253 (67.8)	15 (60.0)	44 (67.7)	164 (70.4)	4 (50.0)	26 (61.9)	0.52
Gastrointestinal bleeding (%)	147 (39.4)	8 (32.0)	26 (40.0)	96 (41.2)	2 (25.0)	15 (35.7)	0.77
Spontaneous bacterial peritonitis (%)	76 (20.4)	5 (20.0)	17 (26.2)	45 (19.3)	1 (12.5)	8 (19.0)	0.76
Hepatocellular carcinoma (%)	23 (6.2)	1 (4.0)	5 (7.7)	12 (5.2)	0 (0.0)	5 (11.9)	0.43
**Characteristics of admission**							
ICU admission (%)	332 (88.8)	23 (92.0)	55 (84.6)	215 (91.9)	4 (50.0)	35 (83.3)	0.002
Admission MAP (mmHg)	66.0 [57.4, 74.0]	66.7 [57.3, 71.3]	70.0 [57.3, 75.0]	63.8 [57.3, 74.0]	80.5 [67.3, 89.0]	67.2 [58.7, 72.2]	0.07
Vasopressor for shock (%)	258 (69.4)	16 (64.0)	39 (61.9)	174 (74.4)	5 (62.5)	24 (57.1)	0.10
Vasoconstrictor for HRS (%)	262 (70.2)	15 (62.5)	53 (81.5)	161 (68.8)	3 (37.5)	30 (71.4)	0.06
Albumin given during admission (%)	320 (86.3)	17 (68.0)	62 (96.9)	201 (86.6)	3 (37.5)	37 (88.1)	<0.001
Total albumin given during admission* (g)	125 [75, 169]	150 [25, 175]	125 [97, 200]	125 [75, 200]	150 [130, 150]	79 [50, 150]	0.22
Mechanical ventilation (%)	231 (61.9)	13 (52.0)	33 (50.8)	164 (70.1)	3 (37.5)	18 (43.9)	0.001
Initial RRT modality (%)							0.04
IHD	108 (29.0)	6 (25.0)	28 (43.1)	58 (24.9)	4 (50.0)	12 (28.6)	
CRRT	264 (71.0)	18 (75.0)	37 (56.9)	175 (75.1)	4 (50.0)	30 (71.4)	
Days from admission to RRT initiation†	4 [1, 9]	3 [1, 16]	5 [2, 11]	4 [1, 8]	5 [4, 9]	4 [2, 9]	0.36
**Survival Scores**							
MELD-Na score	32 [25, 37]	31 [22, 34]	33 [27, 35]	31 [24, 37]	27 [22, 36]	33 [25, 36]	0.49
CLIF-C ACLF score	56.1 [49.1, 62.5]	49.5 [42.9, 56.0]	53.6 [47.3, 61.5]	57.0 [51.4, 63.1]	54.1 [44.6, 59.1]	56.8 [48.1, 60.6]	0.01

**Laboratory values**							
Sodium (mEq/L)	133 [128, 137]	133 [131, 137]	132 [127, 137]	133 [129, 137]	139 [134, 140]	131 [127, 137]	0.19
Admission creatinine (mg/dL)	2.5 [1.5, 3.9]	2.1 [1.3, 2.6]	2.8 [1.9, 3.9]	2.4 [1.4, 3.9]	2.4 [1.8, 4.1]	2.9 [1.8, 4.4]	0.13
Peak creatinine (mg/dL)	4.0 [2.9, 5.9]	2.7 [2.1, 3.7]	4.1 [3.2, 5.8]	4.2 [3.0, 6.0]	4.2 [3.2, 6.8]	4.4 [3.0, 6.4]	0.001
BUN (mg/dL)	44 [28, 65]	39 [23, 63]	52 [39, 73]	40 [24, 60]	55 [51, 65]	58 [32, 84]	0.001
White blood count (K/uL)	10.1 [6.7, 16.0]	7.3 [5.5, 11.6]	10.7 [7.7, 16.9]	10.5 [7.1, 15.9]	7.8 [5.1, 15.0]	9.4 [5.3, 15.8]	0.12
Albumin (g/dL)	2.9 [2.4, 3.3]	2.9 [2.5, 3.4]	2.9 [2.5, 3.5]	2.8 [2.4, 3.2]	3.3 [2.9, 3.6]	2.8 [2.6, 3.4]	0.36
International normalized ratio (INR)	2.0 [1.5, 2.7]	2.0 [1.5, 2.4]	1.9 [1.6, 2.4]	2.0 [1.5, 2.7]	1.7 [1.4, 2.7]	1.9 [1.4, 2.6]	0.81
Total bilirubin (mg/dL)	6.2 [2.2, 16.8]	3.4 [2.0, 9.7]	6.6 [3.1, 15.6]	6.4 [2.2, 18.3]	6.7 [1.0, 25.9]	6.4 [0.9, 12.7]	0.34
FENa‡ (%)	0.3 [0.2, 0.7]	0.2 [0.2, 0.5]	0.3 [0.1, 0.5]	0.4 [0.2, 1.0]	0.2 [0.2, 0.7]	0.4 [0.2, 0.6]	0.24

Continuous variables are reported as median [interquartile range].

* Available in 272 patients.

† Available in 333 patients.

‡ Available in 229 patients.

Abbreviations: MASLD, Metabolic dysfunction-associated steatotic liver disease; ICU, intensive care unit; MAP, mean arterial pressure; HRS, hepatorenal syndrome; RRT, renal replacement therapy; IHD, intermittent hemodialysis; CRRT, continuous RRT; BUN, blood urea nitrogen; FENa, fractional excretion of sodium.

Supplementary Table S4: Demographics and clinical characteristics of the study cohort by liver transplant listed status

Parameter	Liver transplant listed		p
	No (n = 275)	Yes (n = 99)	
**Demographics**			
Age (years)	59 [49, 66]	56 [46, 65]	0.11
Female sex (%)	118 (42.9)	42 (42.4)	0.99
White race (%)	198 (72.0)	73 (73.7)	0.84
Hispanic ethnicity (%)	30 (10.9)	13 (13.1)	0.68
**Comorbidities**			
Etiology of cirrhosis (%)			0.55
Alcohol	121 (44.0)	35 (35.4)	
Hepatitis C	26 (9.5)	9 (9.1)	
MASLD	49 (17.8)	23 (23.2)	
Multifactorial	21 (7.6)	7 (7.1)	
Other	58 (21.1)	25 (25.2)	
Etiology of AKI (%)			<0.001
Prerenal AKI	17 (6.2)	8 (8.1)	
HRS-AKI	34 (12.4)	31 (31.3)	
Acute tubular necrosis	188 (68.4)	46 (46.5)	
Other AKI	8 (2.9)	0 (0.0)	
Unable to classify	28 (10.2)	14 (14.1)	
Diabetes (%)	94 (34.2)	36 (36.4)	0.79
Coronary artery disease (%)	54 (19.6)	4 (4.0)	<0.001
Chronic kidney disease (%)	67 (24.4)	23 (23.5)	0.97
Hypertension (%)	137 (49.8)	33 (33.3)	0.007
**Complications of cirrhosis**			
Ascites (%)	227 (82.5)	90 (90.9)	0.05
Encephalopathy (%)	178 (65.0)	75 (75.8)	0.07
Gastrointestinal bleeding (%)	106 (38.7)	41 (41.4)	0.72
Spontaneous bacterial peritonitis (%)	53 (19.3)	23 (23.2)	0.50
Hepatocellular carcinoma (%)	16 (5.8)	7 (7.1)	0.63
**Characteristics of admission**			
ICU admission (%)	241 (87.6)	91 (91.9)	0.33
Admission MAP (mmHg)	66.0 [57.5, 74.0]	65.3 [57.5, 74.2]	0.95
Vasopressor for shock (%)	191 (69.5)	67 (69.1)	0.99
Vasoconstrictor for HRS (%)	180 (65.5)	82 (83.7)	0.001
Albumin given during admission (%)	225 (82.4)	95 (96.9)	<0.001
Total albumin given during admission* (g)	125 [75, 200]	125 [80, 175]	0.83
Mechanical ventilation (%)	175 (63.9)	56 (56.6)	0.25
Initial RRT modality (%)			0.81
IHD	81 (29.6)	27 (27.6)	
CRRT	193 (70.4)	71 (72.4)	
Days from admission to RRT initiation†	4 [1, 9]	5 [2, 11]	0.09
**Survival Scores**			
MELD-Na score	31 [24, 36]	33 [29, 37]	0.04
CLIF-C ACLF score	56.6 [49.7, 62.8]	55.1 [47.8, 60.6]	0.07
**Laboratory values**			
Sodium (mEq/L)	134 [129, 138]	131 [124, 137]	0.001
Admission creatinine (mg/dL)	2.4 [1.4, 4.0]	2.5 [1.7, 3.9]	0.51
Peak creatinine (mg/dL)	4.0 [2.9, 5.8]	4.1 [2.8, 5.9]	0.85
BUN (mg/dL)	40 [24, 64]	48 [37, 68]	0.004
White blood count (K/uL)	10.4 [6.9, 16.3]	9.6 [6.5, 14.8]	0.23
Albumin (g/dL)	2.9 [2.4, 3.3]	2.9 [2.5, 3.3]	0.53
International normalized ratio (INR)	2.0 [1.5, 2.7]	1.9 [1.6, 2.4]	0.41
Total bilirubin (mg/dL)	5.1 [1.9, 15.5]	8.2 [3.3, 19.6]	0.006
FENa‡ (%)	0.3 [0.2, 0.9]	0.3 [0.2, 0.6]	0.63

Continuous variables are reported as median [interquartile range].

* Available in 272 patients.

† Available in 333 patients.

‡ Available in 229 patients.

Abbreviations: MASLD, Metabolic dysfunction-associated steatotic liver disease; AKI, acute kidney injury; HRS, hepatorenal syndrome; ICU, intensive care unit; MAP, mean arterial pressure; RRT, renal replacement therapy; IHD, intermittent hemodialysis; CRRT, continuous RRT; BUN, blood urea nitrogen; FENa, fractional excretion of sodium.

Supplementary Table S5: Multivariable Fine-Gray subdistribution hazard sensitivity analysis for 90-day mortality in patients with cirrhosis and AKI-RRT (HRS-AKI vs. ATN)

**Parameter**	**Fine-Gray subdistribution hazard regression model**		
	**sHR**	**95% CI**	**p**
Etiology of AKI: HRS-AKI (vs. ATN)	1.14	0.77 – 1.69	0.51
Age (per 1-year)	1.00	0.99 – 1.02	0.45
Sex: Female (vs. Male)	0.91	0.66 – 1.25	0.55
Race: White (vs. non-White)	1.19	0.83 – 1.70	0.33
Ethnicity: Hispanic (vs. non-Hispanic)	0.74	0.46 – 1.21	0.23
Volume capacity: Non-high (vs. High)	1.39	1.00 – 1.94	0.05
Vasopressor for shock: Yes (vs. No)	1.26	0.86 – 1.83	0.23
Initial RRT modality: CRRT (vs. IHD)	2.57	1.74 – 3.80	<0.001
MELD-Na score (per 1-point)	1.04	1.02 – 1.06	<0.001
Liver transplant: Listed (vs. non-listed)	0.21	0.12 – 0.36	<0.001

Abbreviations: AKI, acute kidney injury; RRT, renal replacement therapy; HRS, hepatorenal syndrome; ATN, acute tubular necrosis; CRRT, continuous RRT; IHD, intermittent hemodialysis.

Supplementary Table S6: Multivariable Fine-Gray subdistribution hazard sensitivity analysis for 90-day mortality in patients with cirrhosis and AKI-RRT, including CLIF-C ACLF score in the model

Parameter*	Fine-Gray subdistribution hazard regression model		
	sHR	95% CI	p
Etiology of AKI: HRS-AKI (vs. non-HRS-AKI)	1.40	0.99 – 1.98	0.06
Sex: Female (vs. Male)	0.98	0.75 – 1.28	0.89
Race: White (vs. non-White)	1.32	0.97 – 1.81	0.08
Ethnicity: Hispanic (vs. non-Hispanic)	0.72	0.46 – 1.11	0.14
Volume capacity: Non-high (vs. High)	1.26	0.93 – 1.71	0.13
Initial RRT modality: CRRT (vs. IHD)	3.11	2.21 – 4.37	<0.001
CLIF-C ACLF score (per 1-point)	1.03	1.02 – 1.05	<0.001
Liver transplant: Listed (vs. non-listed)	0.21	0.14 – 0.34	<0.001

* The variables age, vasopressor for shock, and MELD-Na score were not included in the model because of collinearity with the CLIF-C ACLF score.

Abbreviations: AKI, acute kidney injury; RRT, renal replacement therapy; HRS, hepatorenal syndrome; CRRT, continuous RRT; IHD, intermittent hemodialysis.
